# Knowledge and attitude to dental implant placement amongst a group of Nigerian dentist

**DOI:** 10.4314/ahs.v22i2.77

**Published:** 2022-06

**Authors:** Joan Enabulele, Julie Omo, Louis Ibhawoh

**Affiliations:** Restorative Dentistry; University of Benin; University of Benin, Restorative Dentistry

**Keywords:** dental implant, Dentist, Knowledge

## Abstract

**Background:**

The perceptions and experiences of dentists from different specialties on the use of dental implant and its applications can be instrumental in its use exhaustively.

**Objectives:**

To determine the opinions and experiences of a population of Nigerian dentists towards dental implant.

**Methods:**

Data for the study were collated using a self-administered questionnaire. The questionnaire assessed the familiarity of the participants with various implant systems, their designs, sizes and loading as well as the provision of replacement for missing teeth.

**Result:**

The estimated functional life of an implant was reported to be less than 5 years by 2.5% of the respondents; 5 to 10 years by 11.3% of the respondents; 10 to 20 years by 26.3% and more than 20 years by 28.7% of the respondents while 31.3% had no idea about the estimated functional life of an implant. Exposure and experience with dental implants were low as only 30.0% of the respondents had attended any dental implant course/training with 95.0% of the respondents expressing the opinion that they did not have enough training in dental implantology.

**Conclusion:**

The exposure and experience with dental implants was low among the respondents.

## Introduction

The provision of Dental Implants (DI) has become a significant treatment modality to restore aesthetics and function in partially as well as completely edentulous patients^1^, introducing a paradigm modification in restorative treatment possibilities for missing teeth^2^,^3^. DI have also received increased acceptance and satisfaction with their use by patients^3^,^4^. They have also been associated with the conservation of adjacent teeth and alveolar bone^5^ unlike the conventional fixed bridge tooth replacement option. With the advent of DI, missing teeth can now be replaced with stable, comfortable, artificial replacements which feel and look natural^6^. The availability of stable anchorage for prosthetic tooth replacement has obviously also expanded the scope to include better treatment options with the success of implants being an important landmark for dentists when reviewing treatment outcomes with patients^7^. Recent advances in implant technology, materials, designs as well as improved surgical protocol and treatment modalities have played significant roles in making the dental implant a predictably successful treatment option^3^,^8^ with the attendant rapid increase in interest from the public for such treatment^3^.

There is an increased prevalence of DI placement with projections ranging from 5.7% in the most conservative scenario to at least 23% by the year 2026 in a study in the United States^9^. Therefore, oral healthcare professionals will increasingly come across patients with DI restorations, provide dental care and maintenance for them or treat new patients seeking implant treatment.

One of the primary concerns of oral health care providers is to impart positive oral health knowledge and behavior to the society^10^ and this can be achieved through clinical work, organized discussions, lectures and oral health education. It has been advocated that dentists must compulsorily have appropriate knowledge of diagnostic and therapeutic options of dental implant care as well as be able to differentiate between low, medium and high-risk situations to enable early referrals^8^.

The need for implant training among dentists has been advocated and these trainings in dental implantology have been proposed to take place at the undergraduate level and also at the postgraduate level, after dentists have attained the basic skills to practice dentistry, as it may be difficult to complete implant dentistry training within the current duration of undergraduate curriculum^3^,^11^. A general feeling among dental practitioners that they did not have enough didactic and clinical exposure during undergraduate training was reported in India8. Furthermore, a previous study showed that residents claimed not to be provided with sufficient information regarding DI during their undergraduate training^6^. The lack of training courses for dentists and patients' economic status has been reported to lead to poor implant results and a negative attitude to implant placement^12^,^13^. Despite the increased pevalence of dental implant placements, access is observed to be overall still very low with prevalence consistently higher among more advantaged groups^9^.

Currently in Nigeria, DI is now taught at undergraduate level in most of the dental schools^14^ with various forms of pedagogical techniques employed to improve teaching^15^ with additional plans to improve undergraduate teaching of dental implantology^14^. However, there is a dearth of information on the perception of dental implant providers themselves^16^ with only a few studies reported in other countries^6^,^16^,^17^. The dentists' knowledge and attitudes toward a treatment modality can significantly influence treatment decision-making and ultimately, “shape” how oral health care is provided and if it becomes the norm^17^. Hence, this study was undertaken to determine the opinions and experiences of a population of Nigerian dentists towards dental implant.

## Method

This was a descriptive cross-sectional study of dentists and dental students. Data for the study were collated using a self-administered questionnaire. The questions were designed based on previously reported studies^6^,^8^,^17^–^20^. Face and content validity were carried out by a senior dentist who provides dental implant treatment. The questionnaire consisted of 37 close-ended questions and 3 open-ended questions. Information on the demographic characteristics of the participants were obtained. Knowledge on dental implant was assessed using 22 questions. Every correct answer was awarded a score of 1 while a score of 0 was given for wrong answers or unanswered questions. The lowest total score obtainable was 0 and the highest score obtainable was 22. The scores were subsequently graded into poor knowledge (scores less than 50%) and good knowledge (scores 50% and above). The questionnaire also assessed the familiarity of the participants with various implant systems, their designs, sizes and loading as well as the provision of replacement for missing teeth. The opinions of the participants regarding dental implant education, their exposure to implant training and placement, as well as their sources of information regarding dental implant were also obtained.

All data collected were screened for completeness, coded and entered into IBM SPSS version 26.0 for analysis. Descriptive statistics of the data was employed. Continuous variables such as age and knowledge score were represented as mean and standard deviation after checking for normality. Categorical variables were described using frequencies and percentages. Test of association was carried out using chi-square and measure of association was carried out using odds ratio where applicable. Statistically significant association was set at p-value < 0.05.

## Results

Out of a total of 90 questionnaires which were distributed, 81 were returned, giving a response rate of 90.0%. However, one questionnaire was incompletely filled. Therefore, only the 80 properly filled questionnaires were utilised for the study.

The age of the respondents ranged from 24 years to 65 years with a median of 33.5years and interquartile range of 11 with 46.3% belonging to the 31–40years age group and 20.0% representing those more than 40 years of age. There was a higher proportion of male respondents (57.5%) compared to female respondents. The most represented group was the House Officers' making up 41.3% while consultants and senior residents made up 16.3% each (Table 1).

**Table 1:** Demographic characteristics of the respondents

Characteristics	Frequency	Percent
Age (years		
≤30	27	33.75
31–40	37	46.25
>40	16	20.00

Gender		
Male	46	57.50
Female	34	42.50

Status		
Consultant	13	16.25
Senior Resident	13	16.25
Junior Resident	21	26.25
House Officer	33	41.25

**Figure 1:**
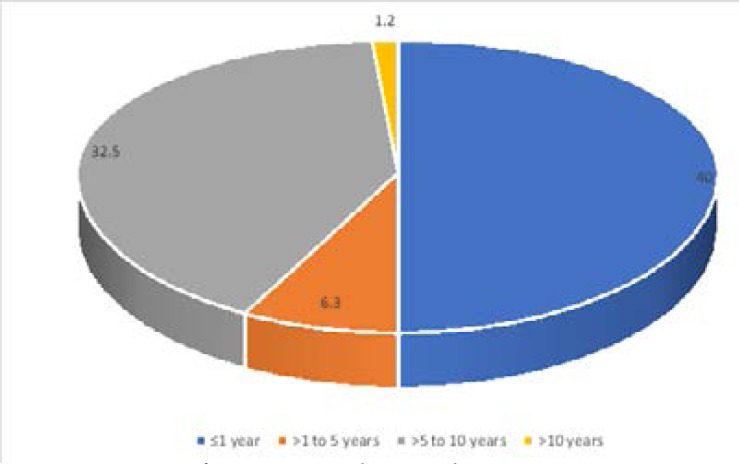
Years of practice among the respondents

The years of practice of the respondents ranged from less than 1 year to 40 years with a median of 6.5 years and interquartile range of 8. Less than a quarter of the respondents (21.3%) had practiced for more than 10 years while 40.0% had practiced for less than a year (Figure 1)

**Figure 2:**
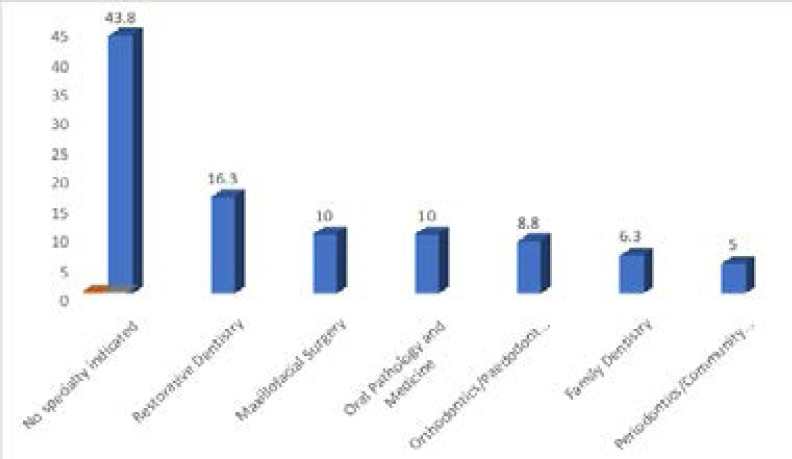
Distribution of respondents by Specialty

Various specialties were represented in this study. Majority (43.8%) of the respondents had not started any form of specialisation or had not decided on which specialty to undertake, while 16.3% were in Restorative Dentistry, 10.0% were in Maxillo-facial Surgery and Oral Pathology and Medicine each. The least represented specialty was Periodontics/Community Dentistry (5.0%) (Figure 2).

With regards to implant placement, only 26.3% of the respondents knew that the safe distance (security zone) between the apical part of implant osteotomy and the inferior alveolar nerve is 2mm. The ideal minimum distance between dental implant and natural tooth of 2mm was correctly stated by 22.5% of the respondents while 17.5% knew that the minimum distance between two implants was 3mm (Table 2).

**Table 2:** Responses with regards to important numbers to know regarding implant placement

Question	Response n=80				
	
	1mm n (%)	2mm n (%)	3mm n (%)	4mm n (%)	No idea n (%)
Safe distance (security zone) between the apical part of implant osteotomy and the inferior alveolar nerve	1 (1.3)	21 (26.3)	15 (18.8)	10 (12.5)	33 (41.3)
Minimum distance between dental implant and natural tooth	4 (5.0)	18 (22.5)	10 (12.5)	10 (12.5)	38 (47.5)
Minimum distance between two implants	3 (3.8)	11 (13.8)	14 (17.5)	11 (13.8)	41 (51.2)

Just less than half (46.3%) of the respondents were of the opinion that dental implants can only be used as fixed prosthetic options while 50.0% stated that dental implants are not only used as fixed prosthetic options while 3.8% of the respondents had no idea regarding this. Majority (80.0%) of the respondents thought that implants were superior to other prosthetic treatment options while 15.0% believed that implants were not superior to other prosthetic treatment options available. 5.0% were unsure if implants were superior to other available prosthetic treatment options. Majority (67.5%) opined that dental implants are equivalent to natural teeth in appearance while 28.7% felt otherwise and 3.8% were uncertain if implants are equivalent to natural teeth in appearance.

**Figure 3:**
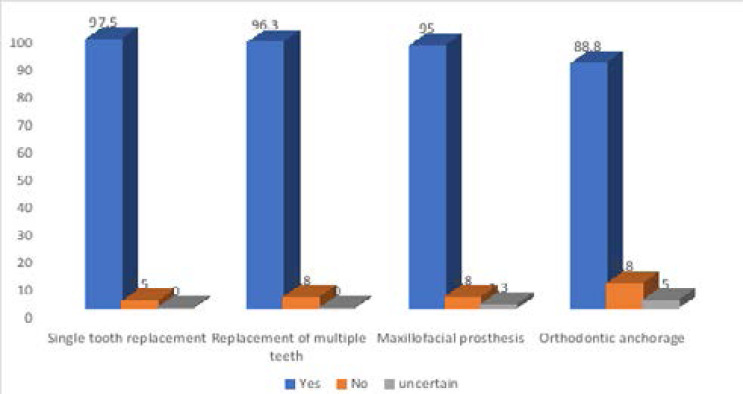
Use of implants among the respondents

With regards to dental implant use, 97.5% of the respondents stated that implants can be used for single tooth replacement; 96.3% opined that implants can be used for the replacement of multiple teeth; 95.0% reported that implants can be used for maxillofacial prostheses and 88.8% recorded that implants can be used for orthodontic anchorage (Figure 3)

Varied responses were received on whether bone resorption after extraction was more in the mandible or in the maxilla, with 38.8% claiming that resorption of bone after extraction is more in the mandible and 26.3% stated that resorption of bone after extraction was more in the maxilla while 35.0% were uncertain which arch suffered more resorption of bone after extraction.

The estimated functional life of an implant was reported to be less than 5 years by 2.5% of the respondents; 5 to 10 years by 11.3% of the respondents; 10 to 20 years by 26.3% and more than 20 years by 28.7% of the respondents while 31.3% had no idea about the estimated functional life of an implant.

Less than half (42.5%) of the respondents knew that dental implants were retained by osseo-integration. Half (50.0%) of the respondents were aware of immediate implant placement with 46.3% of them correctly defining immediate implant placement as placement of an implant immediately or soon after a tooth extraction. Only 26.3% of the respondents claimed to be aware of the different loading protocols. Less than half (42.5%) of the respondents knew that following immediate loading of dental implant, the prosthesis delivered should be temporary, 6.3% claimed the prosthesis should be the final prosthesis while 31.3% stated it could be either the temporary or final and 19.9% were uncertain what the prosthesis should be. Less than half (46.3%) of the respondents claimed to be aware of additional surgical procedures performed to enhance successful implant placements such as bone grafting and sinus lift procedure while 31.3% of them stated the additional surgical procedures correctly.

With regards to limitations to implant therapy, 65.0% stated that cost of the implant was the most limiting factor while 8.8% felt it was the least limiting factor. A dental implant patient's systemic condition was reported to be the most limiting factor by 31.3% of the respondents and 21.3% felt it was the least limiting factor. 5.0% of the respondents reported that “local oral factor(s)” was (were) reported as the most limiting factor by while 62.5% felt it was the least limiting factor.

Table 3 depicts the level of familiarity with dental implant systems, designs, sizes and loading. Less than half (40.0%) of the respondents were somewhat familiar with the different dental implant systems while 33.8% were unfamiliar. A higher proportion (37.5%) of the respondents were unfamiliar with the different dental implant designs while 33.8% were somewhat familiar. Similarly, 35.0% of the respondents were unfamiliar with the different dental implant sizes while 32.5% claimed to be somewhat familiar. One-quarter (25.0%) of the respondents were somewhat familiar with the different dental implant loading techniques while 43.8% were unfamiliar. Exposure and experience with dental implants were low as only 30.0% of the respondents had attended any dental implant course/training with 95.0% of the respondents expressing the opinion that they did not have enough training in dental implantology. Only 17.5% of the respondents had observed any implant surgery/placement and 8.8% had participated in any implant surgery/placement. Only 5.0% of the respondents reported performing implant surgery/placement under supervision while 3.8% claimed to have performed implant surgery/placement without supervision and only 1.3% had placed prosthodontics restoration on an implant. More than half (52.5%) believed that if they are given the opportunity, they will be able to successfully place a dental implant. Almost all (92.5%) of the respondents reported having provided treatment for patients with missing teeth and 82.5% of them claimed to have presented implant as a treatment option to patients.

**Table 3:** Level of familiarity of respondents with Dental Implant Systems, designs, sizes and loading

Familiarity with	Level of familiarity				
	
	Very familiar	Familiar	Somewhat familiar	Unfamiliar	Very unfamiliar
Different dental implant systems	2 (2.5)	10 (12.5)	32 (40.0)	27 (33.8)	9 (11.3)
Different dental implant designs	1(1.3)	12 (15.0)	27 (33.8)	30 (37.5)	10 (12.5)
Different dental implant sizes	2 (2.5)	13 (16.3)	26 (32.5)	28 (35.0)	11 (13.8)
Different dental implant loading	3 (3.8)	13 (16.3)	20 (25.0)	35 (43.8)	9 (11.3)

More than half (53.8%) of the respondents felt that dental implantology should be made into a separate specialty while 41.2% felt it should not and 5.0% were undecided. With regards to the level of education at which dental implantology training should be imparted, 31.3% opined it should be included in the undergraduate curriculum only, 41.2% stated postgraduate curriculum only while 47.5% reported it should be part of both undergraduate and postgraduate curriculum.

**Figure 4:**
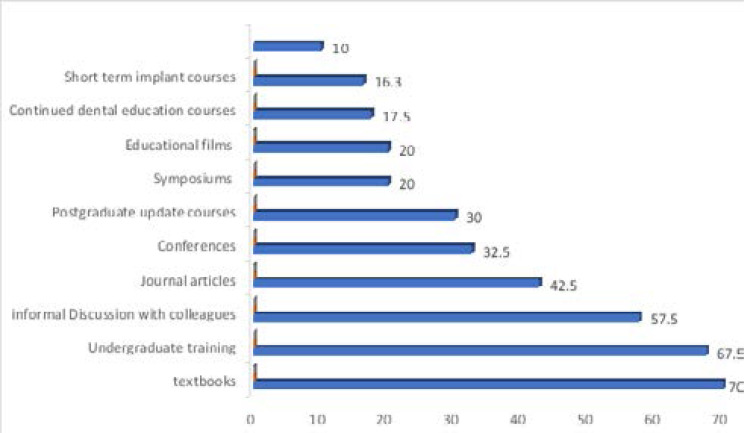
Sources of information regarding dental implantology among the respondents

Various sources of information regarding dental implantology were reported by the respondents with textbooks and undergraduate training reported by 70.0% and 67.5% respectively. The least reported sources were short term implant courses and didactic postgraduate lectures accounting for 16.3% and 10.0% respectively (Figure 4)

Table 4 shows the relationship between knowledge of dental implants among the respondents and sociodemographic characteristics of the respondents. A statistically significant association was observed between the grade of knowledge and specialty of the respondents with a higher proportion of those in Restorative dentistry (76.9%) having ‘good’ knowledge (P=0.04). The gender of the respondents did not have any significant effect on the knowledge of implant dentistry among the respondents (P= 1.00). There was increased knowledge with increasing age of the respondents, but this was not statistically significant (P=0.06). The years of practice had a significant effect on the knowledge of dental implant with an increase in proportion of the respondents having good knowledge with increase in years of practice. Hence a higher proportion of those who had practiced for more than 10 years(76.5%) exhibited good knowledge (P=0.001). The status of the respondents was also observed to have a significant effect on knowledge of dental implant among the respondents with a higher proportion of senior residents (76.9%) having good knowledge followed by consultant (69.2%) (P=0.001). Whether or not a respondent had attended an implant course/training also had a significant effect on knowledge of dental implant among the respondents with a higher proportion of those who had attended an implant training/course (62.6%) having good knowledge (P=0.01). The odds of having good knowledge of dental implant was 3.52 times higher in those who had attended an implant course/training than in those who had not (Odds ratio: 3.52 (1.30–9.55).

**Table 4:** Relationship between the knowledge of dental implants and socio-demographic characteristics of the respondents

Characteristics	Knowledge of Implant dentistry		Total

	Poor	Good	
Gender			**P=1.00**
Male	27 (58.7)	19 (41.3)	48 (100.0)
Female	20 (58.8)	14 (41.2)	34 (100.0)

Age group (years)			**P=0.06**
≤ 30	20 (74.1)	7 (25.9)	27 (100.0)
31–40	21 (56.8)	16 (43.2)	37 (100.0)
>40	6 (37.5)	10 (62.5)	16 (100.0)

Specialty			P=0.04*
Restorative Dentistry	3 (23.1)	10 (76.9)	13 (100.0)
Periodontics/Community Dentistry	2 (50.0)	2 (50.0)	4 (100.0)
Maxillofacial surgery	5 (62.5)	3 (37.5)	8 (100.0)
Orthodontics/Paedodontics	3 (42.9)	4 (57.1)	7 (100.0)
Family Dentistry	4 (80.0)	1 (20.0)	5 (100.0)
Oral Pathology/Medicine	4 (50.0)	4 (50.0)	8 (100.0)
No specialty	26 (74.3)	9 (25.7)	35 (100.0)

Years of practice			**P=0.001**
≤ 1	26 (81.3)	6 (18.8)	32 (100.0)
>1 to 5	3 (60.0)	2 (40.0)	5 (100.0)
>5 to 10	14 (53.8)	12 (46.2)	26 (100.0)
>10	4 (23.6)	13 (76.4)	17 (100.0)

Status			**P=0.001**
Consultant	4 (30.8)	9 (69.2)	13 (100.0)
Senior resident	3 (23.1)	10 (76.9)	13 (100.0)
Junior resident	16 (76.2)	5 (23.8)	21 (100.0)
House officer	24 (72.7)	9 (27.3)	33 (100.0)

Attended implant course			**P=0.01**
Yes	9 (37.5)	15 (62.5)	24 (100.0)
No	38 (67.9)	18 (32.1)	56 (100.0)

Total	47 (58.8)	33 (41.2)	80 (100.0)

## Discussion

The use of dental implants in the restoration of edentulism is gaining more popularity and its knowledge and applications cannot be over emphasized. The perceptions and experiences of different cadres of dentists from different specialties on its use and applications can be instrumental to advocating this emerging form of tooth replacement in their treatment planning interactions with their dental patients.

The age range of 24 to 65 years of age in this study covers the different cadres of doctors ranging from House Officers to consultants, with a majority belonging to the 31 to 40 years age group. There was a male preponderance in this study, a similar finding reflected in some studies^12^,^16^, but not in another^21^.

This study was carried out among dental practitioners across various specialties and cadres, with Restorative dentistry being the most represented specialty although many of the participants (House Officers cadre) were yet to go into specialization. However, in a similar study^12^, most of the respondents were from the Maxillo-facial surgery specialty.

Damage to the inferior alveolar nerve is the most frequently encountered complication in implant surgery^22^. It is an unpleasant experience for patients that could result in mild paraesthesia or complete anaesthesia and this tend to affect their everyday actions such as speech, eating, drinking and other activities^23^. When placing implant, it is safe to leave a 2mm safe zone between the apical part of the implant and the inferior alveolar nerve. From this study however, only a little above a quarter of the participants reported knowing this. Dental implants can either be used as treatment for as a fixed or removable prosthesis. It was observed in this study that only half of the respondents were of this opinion, while the other half either thought it could only be useful in the fixed prosthetic option or had no idea. The reason for the lack of knowledge could be because dental implant practice was not common among the respondents.

Majority of the respondents thought that implant restorations were superior to other prosthetic treatment options available. Dental implants are becoming the standard of care for missing teeth in many situations^24^; hence they appear to be superior to other prosthetic options of restoration depending on patient local and systemic factors. This observation is in concord with that of a study^25^ and at variance with another study conducted among health workers where credence was given to other forms of prosthetic restorations^21^. The reason for this disparity may be the population type where the respondents were other allied health workers who may not be directly involved with patient management.

Most of the participants had a good knowledge of the use of dental implant, as they reported that they can be used for single tooth replacement, for the replacement of multiple teeth, for the retention of maxillo-facial prosthesis and for Orthodontic anchorage. In 1989, Misch proposed five prosthetic options for the use of implants, which may replace partial or total dentition^26^. The implant-supported prosthesis is an option for maxillofacial prostheses where they serve for support as well as for retention^27^. Osseo-integrated implants have been used as a valuable adjunct for orthodontic treatment when there is a need for anchorage because they serve as ankylosed teeth and are incapable of being moved by orthodontic forces^28^.

After dental extractions, the alveolar bone undergoes a remodeling process resulting in horizontal and vertical resorption^29^. It has been reported that the resorption of alveolar bone is more in the mandible than in the maxilla because of the smaller surface area of the lower jaw. The maxilla is less resorbed because of its anatomical shape; hence it can provide a better guidance for bite force^30^. The observation in this study showed that the participants had varied responses with only a little more than a quarter of the participants claiming that resorption was more in the mandible.

While the functional longevity of carefully planned and placed dental implants is generally considered to be upwards of 25 years, less than half of the respondents believed that dental implants lasted 20 years or less, while under a third of the respondents had no idea of what the estimated functional life span of the dental implant was. This is important because this is a factor to be considered advantageous in discussions with patients during treatment planning as the other fixed option to implants, conventional fixed prosthetic dentures or bridge replacements, for lost teeth have been reported by other researchers to have mean lengths of service of 6.1 years^31^, 10.5 years^31^ and up to 16.0 years^33^, although no apparent relationship between longevity and the spans of the bridge prostheses was found by other researchers^34^.

Knowledge that dental implants were anchored to bone by osseo-integration was low, even though the successful management of implant dentistry depends on successful osseointegration among other factors^35^. Immediate implant placement after tooth extraction with early loading has become common^36^ hence, not surprising that half of the respondents were aware of it, with some of them correctly defining immediate implant placement as the placement of an implant immediately after or soon after a tooth extraction.

Various implant loading protocols which include immediate, early and late loading have been reviewed in the literature with each having its own shortcomings^37^. Awareness regarding the different loading protocols and the temporary nature of prosthesis delivered following immediate loading of a dental implant was low.

Knowledge of additional surgical procedures to enhance successful implant placements such as sinus lift procedure and bone grafting which^38^, concluded was necessary in more than half of sextants studied was low in this study The high level of unawareness of these implant-related facts imply that consultations by these dental practitioners with intending dental patients, may not provide correct answers to key questions which such patients may ask.

Implant technology has become the “gold standard” of care^39^, but it is limited by cost which is one its greatest barriers^40^. As regards limitations to implant therapy, most of the respondents considered this to be a major limitation, an observation which corroborates that of other studies^25^,^40^. However, others thought that patients' local and systemic factors were the limitations.

Of familiarity with dental implant systems, designs, sizes and loading, less than half of the respondents showed reasonably low familiarity of respondents with the different implant sizes and the different implant loading options which were indicative of unawareness of important information about implant systems. Familiarity of dental practitioners with the currently available implant systems and the ideal clinical circumstances which indicate their best use will provide information which patients can rely on in making implant treatment choices and increase their readiness to accept implant treatment option.

Exposure and experience with dental implants was very low among the respondents, as only slightly more than a quarter of them had attended implant courses or received implant training, with most of them claiming not to have received enough dental implant training. Less than a quarter had observed and participated in implant surgery or placement with only 5.0% having performed implant surgery or placement with or without supervision. This observation is like the findings of another Nigerian study^12^ and at variance with that of a study conducted among Hong Kong general dental practitioners where more than half of the respondents practiced implant dentistry6. The reason given for that observation was that because dental implantology was just beginning to gain popularity in Nigeria and was not then traditionally taught in dental schools, dentists felt uncomfortable using them as a form of restoration^12^. More than half of the respondents felt if given the opportunity in the right circumstance, they would be able to place an implant successfully, a notion shared by respondents in the referenced study^12^. Dentists in this study were willing and ready to place implants if properly trained.

Almost all the respondents had provided treatment for missing teeth and most of them claimed to have mentioned implant as a treatment option. Missing teeth can be restored with replacement implants, fixed bridges or removable dentures and it a good practice to always present the various options to patients giving the factually correct pros and cons of each option.

It has been reported that implant education be included in the dental school curricula in Nigeria^12^ to increase the knowledge and proficiency of dental implantology. A notable observation in this study was that more than half of the respondents opined that dental implantology should be made into a separate specialty, while a little less than half were of the opinion it should be included in both undergraduate and post graduate curricula. It will be good to start off by including dental implantology into the undergraduate and postgraduate curricula to gain proficiency, thereafter it can be made into a specialty of its own.

The various sources of information regarding dental implantology were reported by the respondents to include textbooks, undergraduate training, short term implant courses and didactic postgraduate lectures. Whether or not the participant had received any implant training made a highly significant difference with respect to the practice of implant dentistry. Those who had received implant training had a positive attitude and large practice as compared to those who did not receive implant training^6^. In this study it was observed that a higher proportion of those who had attended an implant training course had fairly good knowledge of dental implantology. A higher proportion of those in Restorative dentistry seemed to have good knowledge about dental implantology. This corroborates findings from other studies where the knowledge of implant dentistry was best among prosthodontists, closely followed by those belonging to the field of Oral and maxillofacial surgery and Periodontics6. Implant dentistry is a multi-disciplinary therapy that comprises surgical and restorative requirements, where the oral surgeons, periodontists, and prosthodontists/restorative dentist are involved in its placement^6^,^12^,^41^. The relatively good knowledge of the Restorative dentists seen in this study could be due to the involvement of the specialty in diagnosis and treatment planning and involvement in the provision of dental implants to patients.

Older respondents appeared to have a better knowledge of dental implants which was in contrast with results from another study where a higher age of the participant had a significant opposing effect on their knowledge of implant dentistry^6^. The reason for the observation in our study could be that the older dentists updated themselves regularly through update courses and continuing dental education.

The years of practice of the respondents had a significant effect on their knowledge of dental implants with an increasingly better knowledge with longer years of practice. Hence, a higher proportion of those who had practiced for more than 10 years exhibited good knowledge. This contrasts with the report of another study where the years of experience had a significant but different effect on the knowledge and practice of implant dentistry. It was observed that those who had <5 years of experience had the best knowledge and a highly positive attitude followed by those with >15 years of experience with majority of them having good knowledge and only 6% of them having a positive attitude whereas those with 5–15 years of experience had the least knowledge and the least positive attitude towards implant dentistry^6^. The undergraduate dental implantology curriculum in Nigeria is yet to be properly developed^12^, most dentists tend to acquire more knowledge about dental implants at the postgraduate level through courses and continuing dental education. The observation that the consultants and senior registrars had better knowledge of dental implants than the other cadres of respondents would support this suggestion that knowledge of dental implants is still mostly acquired at postgraduate level. The reasonably low awareness of participants in this study on the pertinent facts on Dental Implantology, means that efforts should be geared towards deepening dental implant training and practice at both undergraduate and post graduate levels.

## Conclusion

The exposure and experience with dental implants was low among the respondents.

## Recommendation

Dental implantology should be included in the undergraduate and postgraduate curricula to intensify the training and practice at both levels.
